# Morphology and phylogeny of *Avicennia marina* (Forssk.) Vierh. in Iran

**DOI:** 10.1371/journal.pone.0352461

**Published:** 2026-07-07

**Authors:** Farrokh Ghahremaninejad

**Affiliations:** Department of Plant Sciences, Faculty of Biological Sciences, Kharazmi University, Tehran, Iran; ICAR-Indian Agricultural Research Institute, INDIA

## Abstract

Mangrove forests along the Iranian coast of the Persian Gulf are valuable both economically and for biodiversity. The main tree species in these forests is *Avicennia marina* (Forssk.) Vierh. The habitats of this species in Iran are separated by several thousand kilometers from other populations worldwide. Although introgressive hybridization is common in this species, no morphological study has evaluated this issue in Iranian populations. This study investigated the morphological variation of Iranian *A. marina* and used sequencing of ITS regions to infer phylogenetic relationships Iranian *A. marina* populations and other *Avicennia* species. Morphological analyses based on pollen grains and internodes have shown differences between *A. marina* populations which is the main species of the mangrove forests of Iran and a new population was identified, which is here described as *A. marina* subsp. *australis*. The phylogenetic analysis based on the ITS region confirmed the monophyly of Iranian populations and secondary analysis of ITS2 has moderate support from the two populations detected in the present study. Divergence time estimates suggest these lineages separated during the Pliocene-Pleistocene transition, likely due to climatic fluctuations and sea-level changes.

## Introduction

Mangrove forests are distributed across tropical and subtropical regions worldwide [1]. These ecosystems are defined by conditions such as high salinity, elevated thermal levels, significant tidal changes, substantial sediment deposition, and oxygen-poor muddy substrates [2]. In addition to their importance for ecosystem biodiversity, mangrove forests provide significant economic benefits to humans. They serve as vital nursery and breeding grounds for various species such as crabs, prawns, mollusks, finfish, birds, and reptiles. A large share of the global fish catch, up to 80%, depends on mangrove ecosystems, supporting food security for coastal communities [3]. In addition, species of this genus also possess medicinal properties and have been traditionally used by local communities to treat various diseases, including diabetes, malaria, rheumatism, asthma, smallpox, and ulcers [4]. The tree species in mangrove forests worldwide belong to the genus *Avicennia*, which is classified under the family Acanthaceae. This genus comprises approximately eight recognized species, including *A. marina*, *A. balanophora* Stapf & Moldenke, *A. bicolor* Standl., *A. germinans* (L.) L., *A. integra* N.C.Duke, *A. officinalis* L., *A. schaueriana* Stapf & Leechm. ex Moldenke, and *A. tonduzii* Moldenke [4].

Environmental conditions suitable for mangrove growth in Iran occur along the southern coastline, stretching approximately 2,500 kilometers along the Persian Gulf, where the Iranian mangrove forests are located [5]. The main mangrove species in this region are *A. marina*, and *Rhizophora mucronata* Lam. [6]. Among these, *A. marina* is the dominant species in Iran’s mangrove ecosystems. In addition, various other plant species are associated with these habitats, including grasses from the Poaceae family such as *Aeluropus lagopoides* (L.) Thwaites, *Cenchrus ciliaris* L., *Cenchrus setigerus* Vahl, and *Eragrostis cilianensis* (All.) Vignolo ex Janch., as well as species from the Polygonaceae family (*Pteropyrum aucheri* Jaub. & Spach) [7,8].

The species *A. marina* is one of the most important members of the genus *Avicennia* because it has a high ability to grow under various climate change conditions and is valuable for the stability of mangrove ecosystems [9]. Introgressive hybridization is common among various *Avicennia* species, making the interspecific and intraspecific taxonomy and relationships within the genus highly complex [10]. In recent decades, researchers have commonly used a combination of DNA barcoding techniques and morphological traits to clarify taxonomic status and to detect introgressive and interspecific hybridization among plant species [6,10,11]. DNA barcoding methods typically use short, conserved regions of DNA to identify species and infer phylogenetic relationships among them [12]. In plants, common DNA barcode regions include ITS, rbcL, matK, and trnH-psbA. Among these, the internal transcribed spacer (ITS) region, particularly ITS2, is widely used due to its high variability, moderate conservation, and informative secondary structure [11, 13–15]. These features make ITS and ITS2 effective for resolving phylogenetic relationships at both interspecific and intraspecific levels. Numerous studies have successfully applied ITS markers to assess genetic variation and evolutionary relationships among closely related plant species [11,16,17]. Therefore, using the ITS region in phylogenetic analysis of *A. marina* populations in Iran provides valuable insights into their genetic structure and evolutionary history [13,18–20].

Many studies have been conducted on the Iranian mangrove forests to gain insights into their genetic diversity and phylogenetic relationships, which are essential for the conservation of these coastal ecosystems.

At the genus level, molecular barcoding techniques using nuclear ITS and chloroplast trnH-psbA sequences have been applied to clarify the phylogenetic relationships within *Avicennia* species. These markers proved effective for species delineation and highlighted the role of nucleotide differences in driving speciation within the genus, providing a useful framework for biogeographic and evolutionary studies of Iranian mangroves [21].

In another study, genetic variation of *A. marina* populations along the Bushehr coastline was analyzed using microsatellite markers. The researchers detected relatively high allelic diversity and moderate levels of heterozygosity. However, positive inbreeding coefficients and deviations from Hardy-Weinberg equilibrium indicated some population structure and reduced genetic variation, especially in central populations likely affected by habitat fragmentation and exploitation [22].

Building on these studies, more detailed landscape genetic analyses applied spatial PCA and Random Forest models to investigate gene flow and spatial genetic structure across southern Iran. Their findings revealed low to moderate genetic diversity accompanied by significant genetic fragmentation and limited gene flow, influenced by environmental and geographic barriers such as humidity, longitude, and altitude [7].

Despite these advances and the relatively high genetic diversity reported, gaps remain in fully understanding how genetic diversity relates to phylogenetic relationships, biogeography, and divergence times of Iranian *Avicennia* species in the mangrove forests of Iran. Therefore, in this study, we focus on the morphological traits and phylogenetic relationships of Iranian *Avicennia* species, addressing the following aims:

(1) To identify the key morphological traits of Iranian *A. marina*; (2) To assess whether there is evidence of a new subspecies of *A. marina* populations in Iran based on morphological traits, particularly pollen grain characteristics; (3) To determine the ancestral origin of *A. marina* in Iran. To answer these questions, we conducted extensive sampling across Iran, examined the morphological characteristics of *A. marina*, and analyzed DNA sequence data from the nuclear ribosomal ITS region.

## Materials and methods

### Sampling

**Fig 1:**
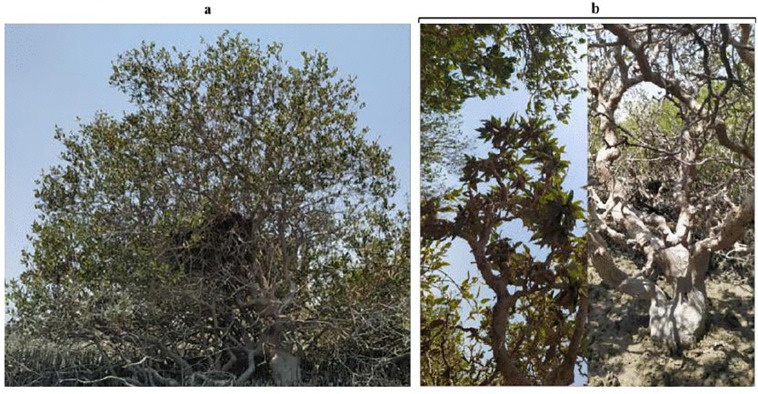
a: Species of *A. marina* subsp. *marina* in the intertidal zones; b: *A. marina* subsp. *australis* subsp. nova, newly described in this research.

**Fig 2:**
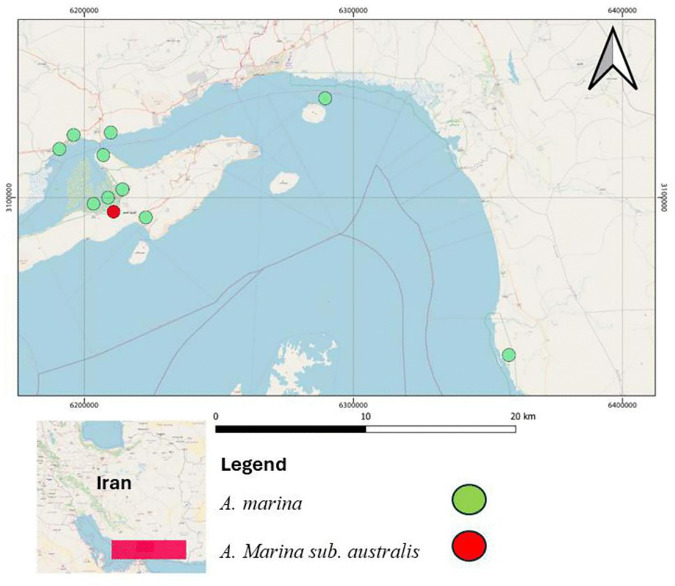
Geographic sampling locations of natural *A. marina* sites in the mangrove forests of southern Iran (Persian Gulf).

To conduct this research, leaf and flower samples of *A. marina* were collected from the main natural habitats of the species in Iran, including the *Harra* (Persian name for *Avicennia*) forests along the Persian Gulf and the Oman Sea (Figs 1 and 2). Sampling permissions were obtained from INSF (Project No. 98021198). All plant samples were morphologically identified by Dr. Farrokh Ghahremaninejad, a specialist in botany at the Department of Plant Sciences, Faculty of Biological Sciences, Kharazmi University. Voucher specimens were deposited in the Tehran Herbarium (T), Kharazmi University under the deposition numbers 2400–2439. Among the sampled populations, one population exhibited distinct morphological traits compared to the others (Figs 1 and 2). This population was later identified and determined as a new subspecies, *A. marina* subsp. *australis*, based on its unique morphological features.

### Morphological measurements

#### Measurement of vegetative and floral traits.

Floral traits were assessed based on mature flowers from selected plants of each population. The following floral characteristics were measured: flower color, pedicel length, corolla lobe shape and size (including petal lobe diameter), and stamen structure. Flower color was described visually, and pedicel length was measured from the flower base to the attachment point on the inflorescence. Petal lobe diameter was measured at the widest point of each lobe, and stamen morphology was examined to determine the degree of connection to the corolla. Microscopic analysis of floral and vegetative structures was performed using a stereomicroscope for initial observations, followed by scanning electron microscopy (SEM) for detailed surface features, particularly those of the floral traits.

### Pollen grains

To examine the micromorphology of pollen grains, the pollen was carefully isolated from different populations under a stereomicroscope. The grains were gently mounted onto special stubs using double-sided adhesive tape and fine forceps. Prior to SEM imaging, the samples were sputter-coated with a thin layer of gold to improve conductivity. The stubs were then placed in a ZEISS EVO MA15 SEM and imaged at selected magnifications.

### DNA extraction and ITS amplification

DNA was extracted from fresh leaves of two populations that showed significant differences in morphological traits, using the CTAB method described previously [23]. The entire ITS regions were amplified with the universal primers ITS-1 and ITS-4 [24]. PCR amplification was performed in 20-μl reaction mixtures using the AccuPower HotStart PCR PreMix kit (Bioneer, Korea). The thermal cycling conditions included an initial denaturation at 95 °C for 360 s, followed by 32 cycles of 60 s at 95 °C, 45 s at 56 °C, 90 s at 72 °C, and a final extension at 72 °C for 5–7 minutes.

Since the universal ITS1 and ITS4 primers can also amplify fungal DNA, two methods were employed to confirm the plant ITS sequences in the samples, as suggested by Jobes and Thien (1997) [25]. In the first method, the amplified ITS was digested with EcoRV, which has a restriction site in the 5.8S sequences of flowering plants (GATATC), before sequencing. After sequencing, the presence of two additional diagnostic markers in angiosperms was checked, including a 14-bp 5.8S rRNA marker (5’ GAATTGCAGAATCC) and a CAAGGAA ITS1 motif from Liu and Schardl (1994). Once confirmed, the PCR products were sent to Bioneer service (Bioneer, Korea; www.Bioneer.com) for sequencing.

### Motif

To identify sequence repeats in the ITS region of *Avicennia* species, we utilized the Repeat Sequence Finder tool available at Novopro Labs (https://www.novoprolabs.com/tools/repeats-sequences-finder). The analysis was performed using the software’s default parameters: length of repeated sequence (2–3 nucleotides), minimum number of repeats (2), minimum length of tandem repeat (5), and percentage of mismatch (0, 2). ITS sequences of various *Avicennia* species were retrieved from public databases and analyzed for the presence of repeat motifs. The dataset included samples from Iran, Brazil, China, Egypt, India, Iraq, the United Kingdom, and the USA.

### Phylogenetic analysis and Secondary structure of ITS2

The electropherograms of the ITS sequences were visually checked using the Chromas software program (version 2.6.6; technelysium.com.au/wp/chromas). Sequence alignment was performed using the MUSCLE method integrated into MEGA 11 software [26]. The nucleotide composition, number of variable sites, parsimony, and conserved sites for all the studied taxa (Two *Avicennia* species from Iran and 21 *Avicennia* species from GenBank (S1 Table)) were calculated separately for the ITS1, 5.8S, and ITS2 regions.

The best-fit nucleotide substitution model for our dataset, identified using MEGA 11, was the Kimura 2-parameter (K2P) model. Phylogenetic relationships were inferred using the Maximum Likelihood (ML) method under the K2P model. Nodal support was evaluated with 1,000 bootstrap replicates [27].

The ITS2 secondary structure and its minimum free energy (ΔG) were predicted through homology modeling, using reference structures from various *Avicennia* species available in GenBank, via the ITS2 Workbench (https://its2.bioapps.biozentrum.uni-wuerzburg.de/).

### Divergence time estimate and biogeographic analysis

Divergence times were estimated by calibrating *Avicennia* species to an average age of 30–35 million years ago (Ma) using a log-normal distribution prior with a standard deviation of 1.0 [6]. Phylogenetic analysis was conducted in BEAST v1.6.1 for 50 million generations, sampling every 5,000 generations. An uncorrelated lognormal relaxed clock model was applied, with the Yule speciation model as the tree prior [28]. The GTR + G substitution model was selected as the best-fit model using jModelTest based on Akaike’s information criterion (AIC). The maximum clade credibility tree was generated using TreeAnnotator v1.7.5 [29] and visualized in FigTree v1.4 [30].

The geographic distribution of *Avicennia* was divided into five regions based on available samples: A (China), B (India), C (USA + Brazil), D (Iran), and E (Saudi Arabia). Biogeographic inferences were conducted using RASP v4.2 software (https://mnh.scu.edu.cn/soft/blog/RASP) [31] with two complementary methods: Statistical Dispersal-Vicariance Analysis (S-DIVA) and Bayesian Binary MCMC (BBM) [32]. S-DIVA reconstructs ancestral areas by statistically optimizing vicariance and dispersal events across a set of trees, thus accounting for phylogenetic uncertainty by analyzing 10,000 trees from the BEAST output along with the maximum clade credibility tree and distribution file. In contrast, BBM uses a Bayesian framework that models biogeographic history as a Markov process, estimating the probabilities of ancestral areas by running MCMC chains under the JC + G (Jukes-Cantor + Gamma) model for 5 million generations; here, only the maximum clade credibility tree and distribution file were used. The combination of these methods allows a robust inference of biogeographic history by integrating different assumptions and analytical frameworks.

## Results

### Morphological results

In this study, a new subspecies of *A. marina* was identified and is here described as *A. marina* subsp. *australis*.

### Avicennia marina (Forssk.) Vierh. subsp. australis F.Ghahrem., subsp. nova

**Fig 3:**
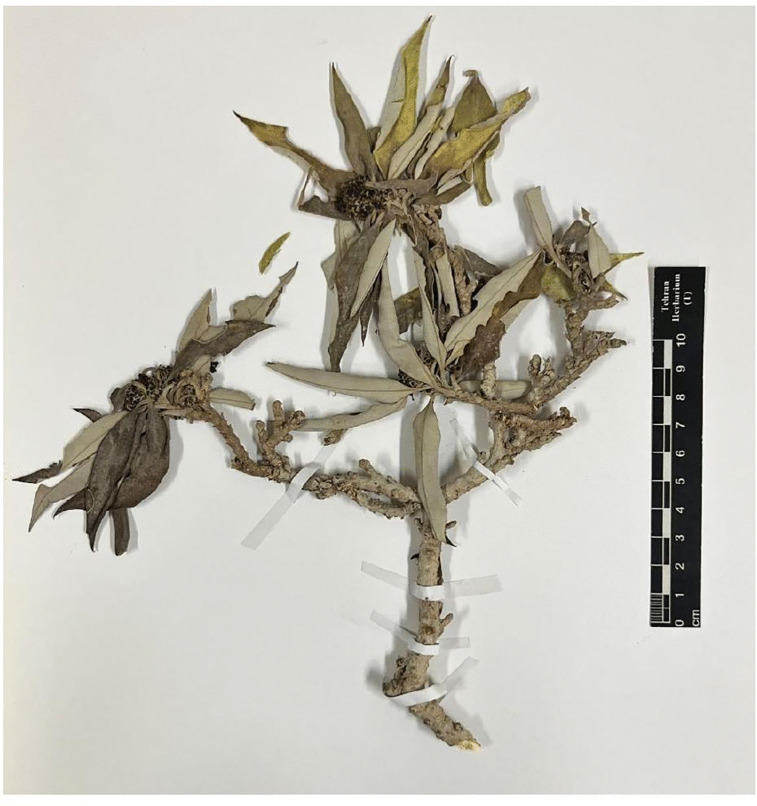
*Avicennia marina* (Forssk.) Vierh. subsp. *australis* F. Ghahrem. subsp. nova; F. Ghahremaninejad & M. Mohammadi 24195 (Tehran herbarium: T), holotype.

**Fig 4:**
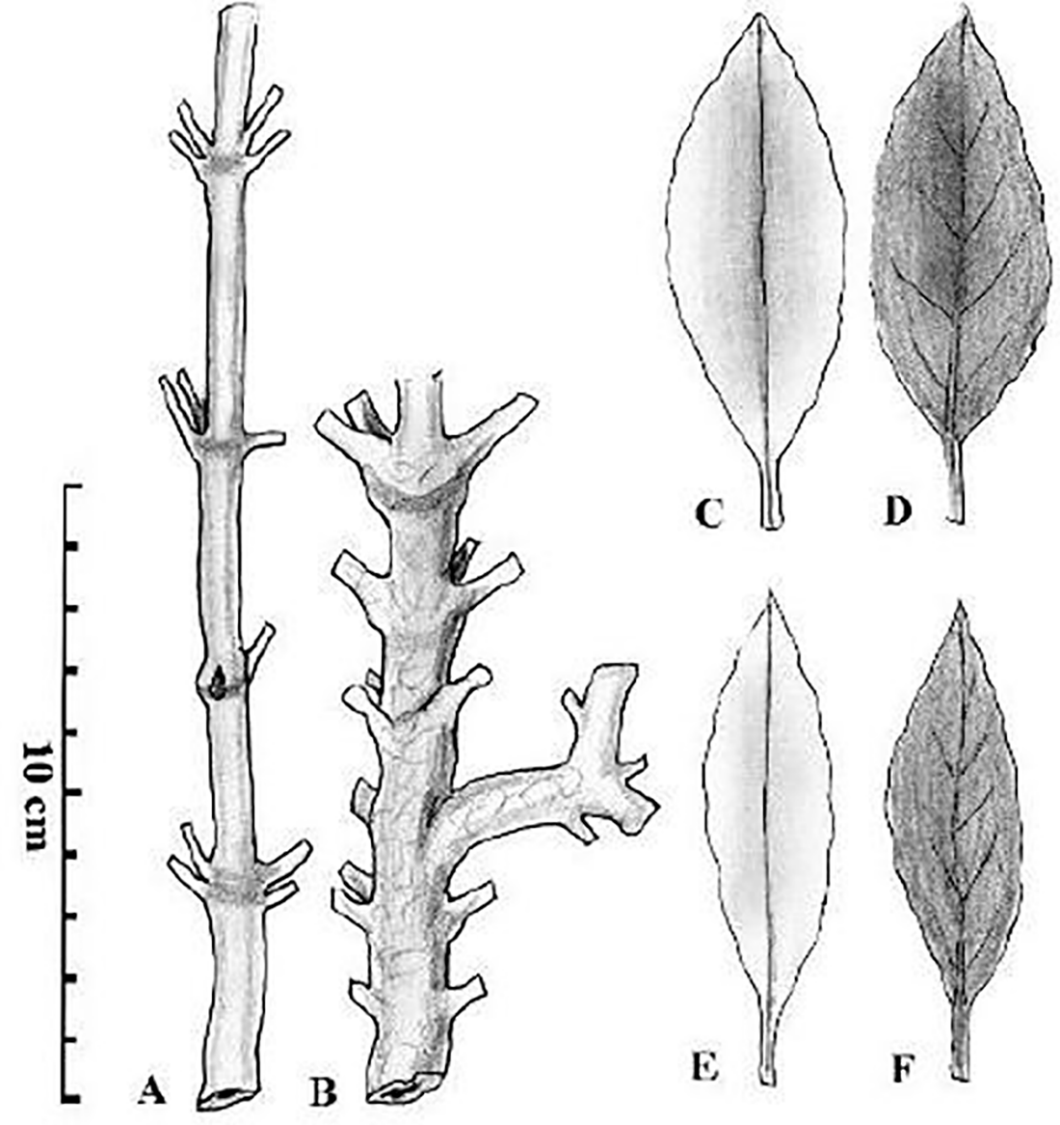
Leaf shape and internode structure of *A. marina* subsp. *marina* and *A. marina* subsp. *australis*, subsp. nova, for comparative analysis (Drawing by H. Nazari). A, C, D: *A. marina* subsp. *marina*; B, E, F: *A. marina* subsp. *australis*.

Shrub, 3–4 m tall. Pneumatophores 18–22 cm long. Internode length ranges from 4 to 7 mm. Leaves are lanceolate to ovate-lanceolate or elliptic, coriaceous, 64–75 mm long, 17–25 mm wide, with acute to acuminate tips and entire margins. The upper surface is glabrous and white-green, often turning blackish when dried. Petioles 6–9 mm long. Flowers yellow to yellow-orange, sessile, and arranged in heads at the apex of short peduncles. Each flower has a concave bract and two bracteoles, ovate to suborbicular, usually shorter than the sepals, and white-ciliate; the bracts are 2.6–3.1 mm long, 1.8–2 mm wide, and acute at the tip. The calyx is 5-partite nearly to the base, with ovate to suborbicular lobes, connate at the base, concave, obtuse, ciliate, and tomentose on the back. The corolla with 4 lobes forming a very short tube at the base. Petal lobes ovate, about 2.8 mm in diameter and approximately 5 mm long (including a ~ 1.1 mm tube). There are 4 sub-sessile stamens, included within the flower and alternating with the corolla lobes. The ovary is elliptic and villous; the style is shorter than the ovary and bifid (2-fid). Fruit unknown. (Figs 3 and 4).

**Typus:** Iran. Hormozgan Province: Qeshm, Mangrove forest of Soheili, 55° 46’ 24.25’‘ E, 26° 47’ 13.19’‘ N, F. Ghahremaninejad & M. Mohammadi 24195 (Holotype: T!; Isotypes: T!).

### Morphological differences between *A. marina* and *A.* *marina* subsp. *australis* in Iran

The morphological comparison of two identified populations of *A. marina* in Iran revealed distinct differences in vegetative and reproductive traits. The main population, *A. marina*, exhibited an internode length ranging from 17 to 106 mm, whereas *A. marina* subsp. *australis* population had a shorter internode length of 4–7 mm. Leaf length varied between 37 and 106 mm in *A. marina*, whereas *A. marina* subsp. *australis* had a narrower range of 64–75 mm. Similarly, leaf width ranged from 10 to 35 mm in *A. marina* and 17–25 mm in *A. marina* subsp. *australis*. Petiole length in *A. marina* was 3–10 mm, whereas in the newly recognized population, it was 6–9 mm.

Floral traits also differed between the two taxa. *A. marina* flowers were dark yellow, while *A. marina* subsp. *australis* flowers ranged from yellow to yellow-orange. Flowers were sessile in *A. marina*, whereas *A. marina* subsp. *australis* had a short pedicel. The corolla of *A. marina* had four nearly unequal, acute lobes, while in *A. marina* subsp. *australis*, the four lobes were at the base and usually equal. Petal lobe diameter measured 5 mm in *A. marina* and 2.8 mm in *A. marina* subsp. *australis*. Both taxa had 4 stamens alternating with the corolla lobes; however, in *A. marina*, they were adnate to the corolla and enclosed within it, whereas in *A. marina* subsp. *australis*, the stamens were semi-connate (Table 1).

**Table 1:** Comparative morphological characteristics of *A. marina* and *A. marina* subsp. *australis* in Iran, highlighting differences in vegetative and floral traits.

Traits	*A. marina*	*A. marina* subsp. *australis*
**Internode Length (mm)**	17–106	4–7
**Leaf Length (mm)**	37–106	64–75
**Leaf Width (mm)**	10–35	17–25
**Petiole Length (mm)**	3–10	6–9
**Flower Color**	Dark yellow	Yellow to yellow-orange
**Pedicel Length (mm)**	Sessile (without pedicel)	Short
**Corolla**	4 lobes; acute; nearly unequal	4 lobes at the base; usually equal
**Petal Lobe Diameter (mm)**	5	2.8
**Stamens**	4; adnate to the corolla and enclosed within it; alternating with the corolla lobes	4; semi-connate; alternating with the corolla lobes

### Pollen Grains

**Fig 5:**
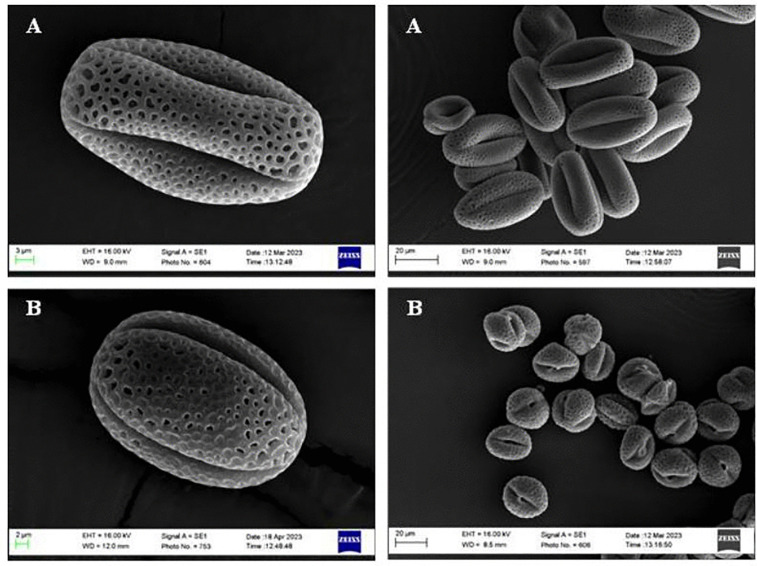
A. Pollen grain of *A. marina*; B. Pollen grain of *A. marina* subsp. *australis.*

Scanning electron microscopy (SEM) analysis of pollen grains from *A. marina*, and *A. marina* subsp*. australis* revealed that their shape is elliptic, with a 3-colporate exine and reticulate exine ornamentation, pollen sizes for *A. marina*, and *A. marina* subsp*. australis* were 16 μm and 21 μm, respectively (Fig 5).

### Characteristics of the ITS Regions in Iranian Avicennia Compared to GenBank Sequences

Our analysis revealed that the ITS1, 5.8S, and ITS2 regions for *Avicennia* samples from Iran were 246 bp, 180 bp, and 231 bp in length, respectively. These lengths closely matched those of *Avicennia* species available in the GenBank database, which are reported as 246 bp, 180 bp, and 231 bp. The most variable regions among the Iranian *Avicennia* samples were identified within the ITS1 and ITS2 regions, which are the same as in other *Avicennia* species from GenBank. Overall, the number of conserved sites across the three nucleolar regions (ITS1, 5.8S, and ITS2) in the Iranian samples was higher than that observed in the GenBank *Avicennia* sequences. This highlights a higher degree of conservation within the Iranian *Avicennia* populations compared to the broader species dataset (Table 2).

**Table 2:** Characteristics of the aligned ITS1, 5.8S, and ITS2 data matrix used for phylogenetic analysis.

	Iranian *Avicennia*			Species *Avicennia* in the GenBank		
Region	ITS1	5.8S	ITS2	ITS1	5.8S	ITS2
**A (%)**	18.38	21.3	17.0	19.33	21.3	17.0
**C (%)**	35.53	31.7	34.5	35.22	31.5	34.5
**G (%)**	32.23	26.3	34.0	31.65	26.4	33.8
**U (%)**	13.84	20.7	14.3	13.73	20.7	14.6
**Length (bp)**	246	180	231	246	180	231
**Conserved site**	237	178	202	161	175	188
**Variable site**	4	–	1	82	4	20
**Parsimony site**	–	–	–	40	2	14

### Motif

The repeat motif analysis revealed significant variation in the presence and distribution of sequence repeats among *Avicennia* species. Among the Iranian samples, *A. marina* exhibited the presence of four repeat motifs (*CCCTCTCCC*, *AAATGCGAT*, *CTCCCGTGC*, and *CCCCGT*), while *A. marina* subsp. *australis* had seven motifs (*CCCTCTCCC*, *AAATGCGAT*, *GACCGCGA*, *TCCCTCGG*, *CTCCCGTGC*, *AACCTGC*, and *CCCCGT*). The unique occurrence of *GACCGCGA* and *TCCCTCGG* in *A. marina* subsp. *australis* suggests a potential variation in repeat patterns between the two Iranian samples.

Compared to other *Avicennia* species, the Iranian *A. marina* shared motifs with samples from China, India, and the USA, particularly *CCCTCTCCC* and *CTCCCGTGC*. However, it lacked certain repeats found in Chinese and Indian samples, such as *GACCGCGA* and *TCCCTCGG*, which were present in *A. marina* subsp*. australis*. This indicates potential subspecies-specific differences in repeat distribution.

Additionally, the motif *AAATGCGAT* was observed in *A. marina* subsp*. australis* from Iran but was absent in the Brazilian and some Chinese samples. The motif *CCCCGT* was shared between Iranian *A. marina* samples and other global populations, including *A. marina* from Egypt and *A. alba* from India. Repeat motif analysis revealed clear differences between *Avicennia* subspecies and geographic populations. Iranian *A. marina* showed four motifs, while *A. marina* subsp. *australasica*from China had seven, including two unique motifs (GACCGCGA and TCCCTCGG), suggesting subspecies-specific variation. Shared motifs among Iranian, Chinese, Indian, and US samples point to conserved elements, whereas unique motifs indicate regional divergence. These repeat motifs may play a role in genome organization or gene regulation, contributing to local adaptation and subspecies differentiation in *Avicennia*. Thus, motif patterns can serve as genetic markers for distinguishing populations and understanding evolutionary processes across the genus (Table 3).

**Table 3:** Analysis of ITS region repeat motifs in *Avicennia* species revealed distinct patterns in Iranian samples, highlighting geographic and subspecies-specific variations.

Position	Country	451- 457	332- 528	61- 657	507-536	142- 497	331- 527	23- 47	128-226	83-172
Motif		CCCTCTCCC	AAATGCGAT	GACCGCGA	TCCCTCGG	AAATGCGAT	CTCCCGTGC	AACCTGC	CCCCGT	CCCGG
**A. *marina***	**Iran**	+	+	–	–	–	+	–	+	–
***A. marina* subsp. *australis***	**Iran**	+	+	+	+	–	–	+	+	+
***A. schaueriana***	**Brazil**	+	–	–	–	–	+	–	+	–
***A. schaueriana***	**Brazil**	+	–	–	–	–	+	+	–	–
**A. *marina* subsp. *australasica***	**China**	+	+	–	–	+	+	+	+	+
***A. marina* var. *rumphiana***	**China**	+	+	–	–	–	–	+	–	+
***A. integra***	**China**	+	–	–	–	+	–	–	–	+
***A. marina***	**China**	+	–	–	–	+	–	–	+	–
***A. marina***	**Egypt**	+	–	–	–	+	–	+	+	+
***A. alba***	**India**	+	+	–	–	–	+	–	+	+
***A. marina***	**India**	+	+	–	–	–	+	+	+	–
***A. officinalis***	**India**	+	–	–	–	+	–	–	+	+
***A. marina***	**Iraq**	+	–	–	–	+	+	–	+	–
***A. marina***	**Saudi Arabia**	+	–	–	–	+	–	–	+	–
***A. bicolor***	**USA**							+	+	–
***A. germinans***	**USA**	+	–	–	–	–	–	+	+	–
***A. marina***	**USA**	+	–	–	–	+	–	+	+	+

### Genetic distance: Kimura 2 parameter

The genetic distance analysis of *Avicennia* species based on the ITS region showed that Iranian *A. marina* populations have a low level of genetic divergence. The mean genetic distance among *Avicennia* species in the present study was calculated as 0.06. The genetic distance between Iranian *A. marina* subsp. *australis* and Iranian *A. marina* ranged from 0.00 to 0.01. Among all comparisons involving Iranian samples, the minimum genetic distance was 0.00 (between Iranian *A. marina* subsp. *australis* and *A. marina*), while the maximum genetic distance was 0.08 (between Iranian *A. marina* and *A. bicolor* from the USA). The mean genetic distance between Iranian *A. marina* and all other *Avicennia* species in the dataset was 0.04. Iranian *A. marina* samples exhibited low genetic distances (0.01–0.03) from populations in China, Egypt, India, Iraq, and the United Kingdom, while they showed higher divergence (0.06–0.08) from American species (*A. bicolor* and *A. germinans*). The genetic distance from Brazilian *A. schaueriana* ranged from 0.06 to 0.07 (Table 4).

**Table 4:** Pairwise genetic distances among *Avicennia* species based on ITS region.

			1	2	3	4	5	6	7	8	9	10	11	12	13	14	15	16
**1**	***A. marina* subsp. *australis***	**Iran**																
**2**	***A. marina***	**Iran**	0.01															
**3**	***A. schaueriana***	**Brazil**	0.07	0.07														
**4**	***A. schaueriana***	**Brazil**	0.07	0.06	0.00													
**5**	***A. marina* subsp. *australasica***	**China**	0.00	0.01	0.07	0.06												
**6**	***A. marina* var. *rumphiana***	**China**	0.02	0.03	0.07	0.07	0.02											
**7**	***A. integra***	**China**	0.03	0.04	0.08	0.08	0.03	0.02										
**8**	***A. marina***	**China**	0.01	0.01	0.07	0.07	0.01	0.03	0.03									
**9**	***A. marina***	**Egypt**	0.01	0.01	0.08	0.07	0.01	0.03	0.03	0.01								
**10**	***A. alba***	**India**	0.00	0.01	0.07	0.07	0.01	0.02	0.03	0.01	0.01							
**11**	***A. marina***	**India**	0.01	0.01	0.07	0.06	0.00	0.02	0.03	0.01	0.01	0.01						
**12**	***A. officinalis***	**India**	0.02	0.02	0.07	0.06	0.01	0.01	0.02	0.02	0.02	0.02	0.01					
**13**	***A. marina***	**Iraq**	0.01	0.01	0.06	0.06	0.00	0.02	0.03	0.01	0.01	0.01	0.00	0.01				
**14**	***A. marina***	**United Kingdom**	0.00	0.01	0.07	0.07	0.01	0.03	0.03	0.01	0.01	0.00	0.01	0.02	0.01			
**15**	***A. bicolor***	**USA**	0.08	0.08	0.02	0.02	0.07	0.07	0.08	0.08	0.08	0.07	0.07	0.07	0.07	0.08		
**16**	***A. germinans***	**USA**	0.07	0.07	0.02	0.02	0.07	0.07	0.08	0.07	0.08	0.07	0.07	0.07	0.06	0.07	0.00	
**17**	***A. marina***	**USA**	0.00	0.01	0.07	0.07	0.01	0.03	0.03	0.01	0.00	0.00	0.01	0.02	0.01	0.00	0.07	0.07

### Secondary structure of ITS2

**Fig 6:**
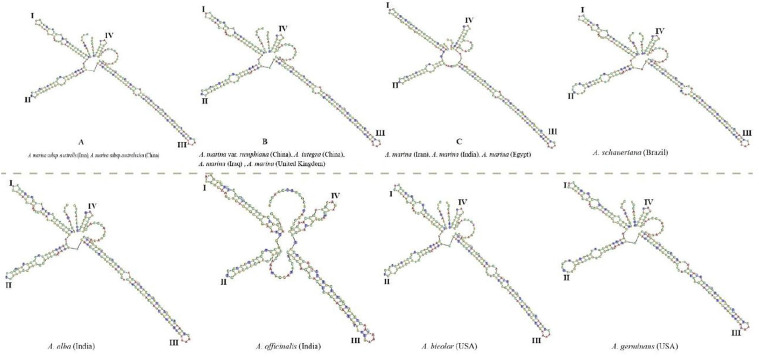
Secondary structure of the ITS2 region in *Avicennia* species. The predicted structures show variations in helix length and unpaired bases among different taxa.

The putative secondary structures of the ITS2 transcript for the studied taxa are shown in Fig 6. A common ITS2 structure [33] was observed in the *Avicennia* genus, consisting of four helices, with Helix III being the longest and a U–U mismatch present in the second helix. The four domains indicate distinct size classes (Table 5).

**Table 5:** Numerical and statistical data of the predicted ITS2 secondary structures analyzed in this study.

		Length (nt) in each helix				Unpaired bases in total
**Taxa**	**Country**	**I**	**II**	**III**	**IV**	
**A**	–	32	32	74	10	71
**B**	–	34	32	74	10	68
**C**	Iran	40	24	74	16	56
***A. schaueriana***	Brazil	34	28	72	12	71
***A. alba***	India	30	32	74	10	73
***A. officinalis***	India	36	22	48	16	92
***A. bicolor***	USA	30	30	70	10	79
***A. germinans***	USA	32	28	70	10	80

**A:** *A. marina* subsp. *australis* (Iran)*, A. marina* subsp. *australasica* (China)- **B:** *A. marina* var. *rumphiana* (China), *A. integra* (China), *A. marina* (Iraq), *A. marina* (Saudi Arabia)- **C:** *A. marina* subsp. *marina* (Iran), *A. marina* (India), *A. marina* (Egypt).

The secondary structure of the ITS2 region among *Avicennia* species exhibited variations in helix length and unpaired bases, reflecting species-specific structural differences. Helix II was the most variable region of ITS2 in *Avicennia* species, typically ranging from 24 to 32 bp. The longest and shortest helices among *Avicennia* species were Helix III and Helix IV, respectively.

Group A (*A. marina* subsp. *australis* from Iran and *A. marina* subsp. *australasica* from China) had relatively similar helix structures, with Helix I ranging from 32 to 34 nt and a total of 68–71 unpaired bases. Group B, which included *A. marina* from Iraq, the United Kingdom, and China, showed slightly more variation, particularly in Helix I (34 nt) and a total of 68 unpaired bases.

The Iranian *A. marina* sample (Group C) exhibited a distinct structural pattern compared to other groups. It had the longest Helix I (40 nt) and the shortest Helix II (24 nt), while Helix III (74 nt) and Helix IV (16 nt) were consistent with most other *A. marina* populations. Importantly, the Iranian sample had the lowest number of unpaired bases (56), suggesting a more stable structure.

Other species outside *A. marina* groups showed more pronounced differences. *A. officinalis* from India had the shortest Helix III (48 nt) and the highest number of unpaired bases (92), indicating a highly flexible RNA structure. *A. schaueriana* (Brazil) and *A. alba* (India) had comparable helix lengths, with total unpaired bases of 71 and 73, respectively. Among the U.S. samples, *A. bicolor* and *A. germinans* exhibited similar helix structures, but *A. germinans* had the highest number of unpaired bases (80).

Overall, the Iranian *A. marina* sample displayed a unique combination of features, particularly its extended Helix I and reduced unpaired bases, which distinguish it from other *A. marina* populations and *Avicennia* species. These differences highlight the potential influence of geographic and environmental factors on ITS2 secondary structure variation (Fig 6).

### Phylogenetic tree

**Fig 7:**
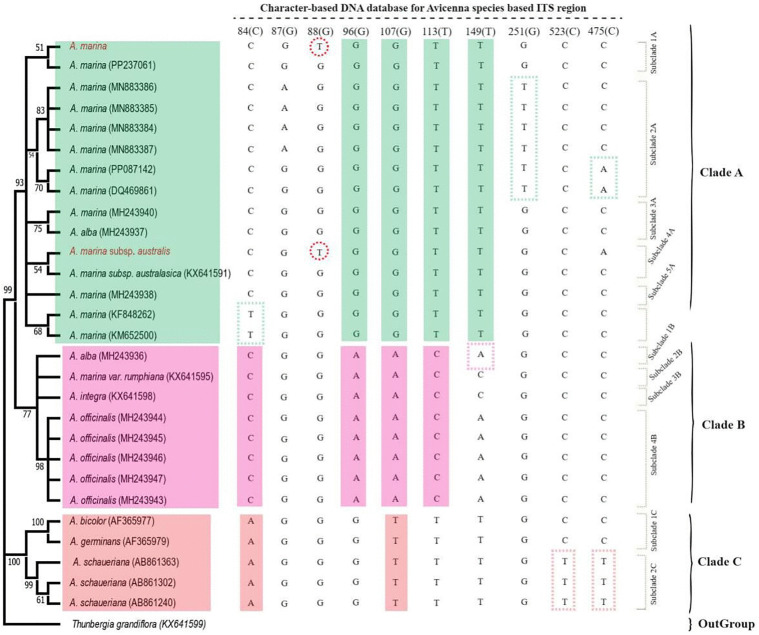
Phylogeny tree maximum likelihood of *Avicennia* species based on ITS region with bootstrap 1000 is reported.

In this study, *Thunbergia grandiflora* was used as the outgroup to confirm the monophyly of the *Avicennia* genus. The clustering pattern highlights a clear separation between *A. marina* and other species, suggesting significant genetic divergence. The phylogenetic tree based on the ITS region of *Avicennia* species revealed three main clades with clear clustering patterns. Clade A primarily includes *A. marina* samples with high bootstrap support values (99%) and is further subdivided into five subclades (1A, 2A, 3A, 4A, and 5A). Subclade 1A groups *A. marina* samples with identical character states at positions 87 (T) and 96 (G). This study revealed that one of the Iranian samples, identified as *A. marina* based on morphological data, is located in subclade 1A along with *A. marina* sample from Iraq. Subclades 2A and 3A exhibit variations at specific sites (523 (C) and 475 (C)). Another sample from Iran, identified for the first time as *A. marina* subsp. *australis* based on morphological data, is located in subclade 4A along with *A.* *marina* subsp. *australasica* samples. Clade B includes *A. alba*, *A. marina* var. *rumphiana*, *A. integra*, and *A. officinalis*. *A. officinalis* samples form a distinct subclade (4B) with strong bootstrap support. This subclade is characterized by variable character states at positions 107 (G) and 149 (T). Variations at positions such as 84 (C) and 87 (G) further distinguish these species from *A. marina*. Clade C comprises *A. bicolor*, *A. germinans*, and *A. schaueriana*, forming a strongly supported group (100%). Subclade 2C is defined by unique substitutions at positions 251 (G) and 523 (T), differentiating these species from Clades A and B. This study also revealed that the nucleotide at position 88 (T) in one of the Iranian samples differs from other species, which have a nucleotide G at this position (Fig 7).

### Divergence Time Estimation

**Fig 8:**
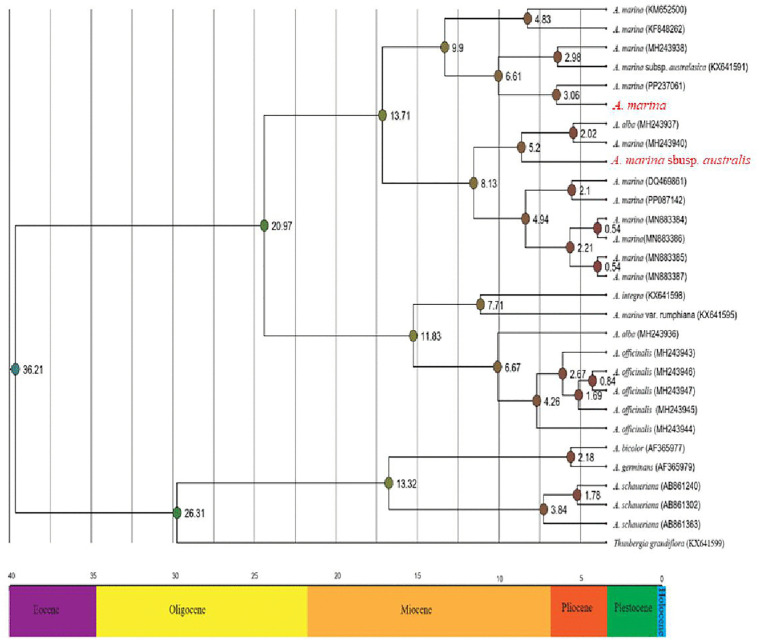
Divergence time estimation of *Avicennia* species based on ITS data. Node ages (Ma) are shown, with colored circles indicating posterior probabilities. The timeline below marks geological epochs.

Divergence time analysis based on the ITS dataset estimated the separation of the *Avicennia* lineage from its closest outgroup, *Thunbergia grandiflora*, at approximately 36.21 Ma during the Eocene. Diversification within *Avicennia* began around 26.31 Ma in the Oligocene, followed by major splits during the Miocene. Iranian *Avicennia* populations in the present study (highlighted in red in Figure 8) form a distinct clade within *A. marina*. Their divergence is estimated to have occurred between 2.96 and 2.02 Mya, during the Pliocene to early Pleistocene. The results indicate that the most recent common ancestor of *A. marina* and its closely related taxa dates back to approximately 13.71 Ma, suggesting significant diversification during the Miocene. Several divergence events occurred between 10 and 5 Ma, leading to the formation of distinct lineages within *A. marina* and other species. The split between *A. officinalis* and *A. germinans* was estimated at 6.67 Ma. The most recent divergences among regional populations of *A. marina* occurred during the Pliocene and Pleistocene (approximately 3–1.7 Ma) (Fig 8).

### Biogeography analysis

**Fig 9:**
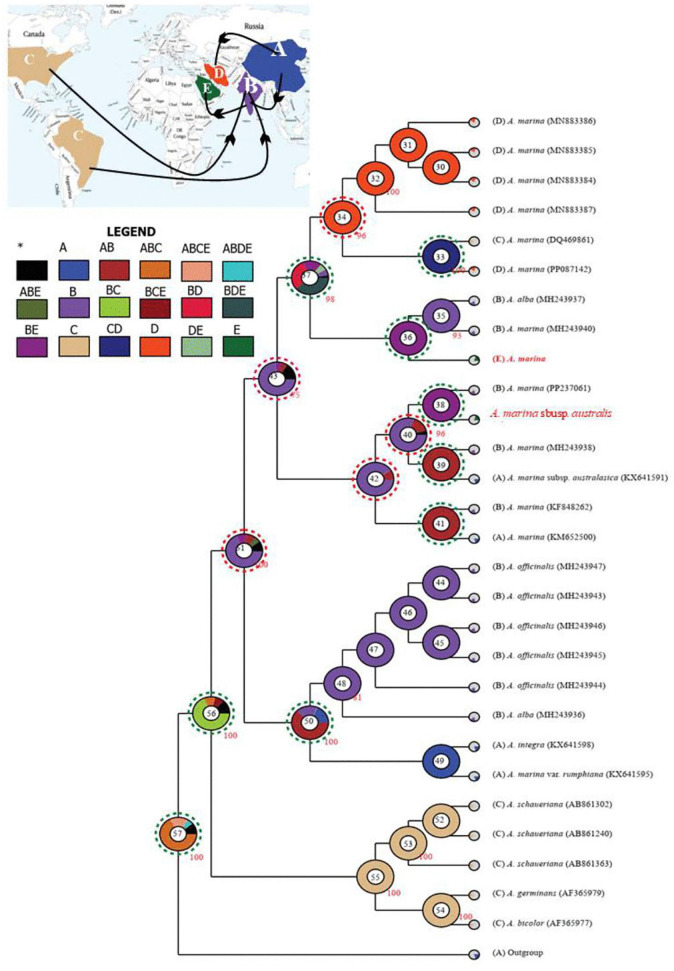
Ancestral state reconstruction of *Avicennia* species based on Statistical Dispersal-Vicariance Analysis (S-DIVA), overlaid on the maximum clade credibility chronogram from BEAST. **(a)** Map showing geographic regions with assigned colors and dispersal routes indicated by red arrows. **(b)** Legend of color codes representing geographic regions used in the analysis. Each color corresponds to one region, consistent across panels: A (China): Dark Blue, B (India): Pastel Orange, C (USA and Brazil): Gold, D (Iran): Orange, E (Saudi Arabia): Dark Green.

The ancestral state reconstruction of *Avicennia* species, based on statistical dispersal-vicariance analysis (S-DIVA), identified multiple dispersal and vicariance events across different geographic regions. The analysis suggests that the common ancestor of *Avicennia* originated in India (B) and with subsequent dispersal to the Middle East (D, E) and the Americas (C). Several nodes indicate high probabilities for dispersal events between these regions. Iranian *Avicennia* species (region D), highlighted in red, form a distinct lineage within *A. marina*. The S-DIVA analysis suggests that these populations originated from ancestral lineages in Asia (A, B), with limited subsequent dispersal. The phylogenetic relationships indicate a unique evolutionary trajectory for Iranian *A. marina*, distinguishing them from other regional populations (Fig 9).

(c) Phylogenetic tree showing evolutionary relationships among *Avicennia* species. Pie charts at each node indicate the relative probability of ancestral distributions, color-coded by region as above. Iranian *A. marina* are highlighted in red font

## Discussion

This study provides insights into the taxonomic status, biogeography, morphological characteristics, and divergence history of *Avicennia* species in the *Harra* forests of Iran. Our results reveal that in the *Harra* forests of Iran, in addition to *A. marina*, which is the main species in this forest, an additional new subspecies named *A. marina* subsp. *australis* was also identified. We identified a new, distinct subspecies of *A. marina*, which was well differentiated from *A. marina* based on both morphological and molecular data. Based on variable sites in the ITS region, only the Iranian sample had nucleotide T at position 88, whereas other species had nucleotide G. This may indicate a closer relationship among Iranian *Avicennia* species compared to other *Avicennia* species worldwide [9]. Another reason that supports the new subspecies *A. marina* subsp. *australis* in the *Harra* forest of Iran is the internode length, which is a diagnostic trait in various species [34,35]. This study demonstrates significant differences between *A. marina* subsp. *australis* and *A. marina*.

### Morphological Characters

Morphological characters are among the main features used to describe new species, and they are often complemented by molecular data [36,37]. The flowers of *Avicennia* species have been described in three main shapes which include ‘germinans’, ‘ofﬁcinalis’ and ‘marina’ [38], one of the main floral character which almost considered by researcher in *Avicennia* species is characteristics of the corolla which shows considerable variation among *Avicennia* species [38], our findings illustrate that the corolla in Iranian *Avicennia* like other *A. marina* which has been reported to have four lobes confirming their placement within *A. marina* [38,39], but in the new subspecies which reported in this corolla is equal at the base whereas *A. marina* is unequal. Another key difference between *A. marina* subsp. *marina* and *A. marina* subsp. *australis* relates to flower color: the former is yellow, while the latter ranges from yellow to orange. Additionally, flower color is recognized as an important morphological characteristic in *Avicennia* species [40].

According to previous studies, the pollen of various *Avicennia* species is divided into two size categories: small (10–20 μm) and medium (above 20 μm) [41]. In the present study, the results showed that the pollen of Iranian *Avicennia* belongs to the small-size category. This finding is similar to previous studies on *A. marina*, which also reported smaller pollen sizes compared to other *Avicennia* species [42]. The size of pollen is one of the key factors in understanding evolutionary processes and species adaptation [43]. However, in *Avicennia* species, the high variability in pollen size makes it difficult to detect ecological patterns and adaptive traits based on this feature [43–45]. In this research, as in previous studies, the pollen size of Iranian *Avicennia* showed a wide range (16–21 µm) [42]. Researchers have identified three pollen grain shapes in *Avicennia* species: prolate, isodiametric, and oblate [41]. In the present study, the Iranian Avicennia pollen was classified as prolate, similar to other *A. marina* populations studied worldwide [42].

### Phylogenetic Insights into *Avicennia* Species

The ITS region shows high polymorphism in the *Avicennia* genus [6], which is considered one of its key characteristics, and also, it is highly conserved within species, making it a suitable DNA marker for species identification (Chen et al., 1992). The ITS region showed five variable positions in Iranian *Avicennia* and 106 in other *Avicennia* species, suggesting high variability within the genus *Avicennia*. The ITS region tends to show high variability in genera where hybridization is common [46], as is the case in the genus *Avicennia*, where interspecific hybridization frequently occurs [10].

### Taxonomic status of *Avicennia* in Iran

*A. marina* is the main species in the *Harra* forests of Iran and is widespread throughout these forests [6,47–49]. Previous studies have shown that *A. marina* is monophyletic, with strong support based on various chloroplast and nuclear DNA regions [6,44,49]. In this study, based on the ITS region and with high clade support, we also found that *A. marina* in Iran is monophyletic. Two samples from the *Harra* forest showed a close relationship with *A. marina* and were placed within *A. marina* clade. The species *A. marina* has a close relationship with *A. alba* and *A. officinalis*, as shown in previous studies, where it formed a sister clade with them. In this research, *A. marina* also formed a sister clade with *A. alba* and *A. officinalis* [50]. The species *A. marina* subsp. *australis* from Iran, in the present study, was placed within *A. marina* clade but formed a sister group with *A. marina* subsp. *australasica* from China. This result suggests the potential presence of an infraspecific taxonomic rank within this species [6].

Phylogenetic relationships of plants have often been studied using DNA sequencing and morphological data. However, in the past decade, the secondary structure of ITS2 has been widely used for phylogenetic reconstruction [51–53]. In this study, we used the secondary structure of ITS2 to investigate the relationships among different *Avicennia* species. We found eight different ITS2 structures in *Avicennia*, which show high variability in this genus. We also identified two ITS2 structures: the first sample was grouped with *Avicennia marina*, and the second sample from Iran had the same structure as *A. marina* subsp. *australasica* from China. This is strong evidence that a new subspecies of *A. marina*, called *A. marina* subsp. *australis*, exists in the *Harra* forest of Iran.

Previous researchers have reported that species diversity is high among the three *Avicennia* species: *A. germinans*, *A. marina*, and *A. schaueriana* [54]. In the present study, we detected the secondary structure of ITS2 and found three different secondary structures among these species and the various varieties of *A. marina*. Additionally, two separate ITS2 secondary structures were identified for *A. germinans* and *A. schaueriana*. These findings support the results of previous studies.

Other *Avicennia* species in this research, including *A. alba*, *A. officinalis*, and *A. bicolor*, showed specific ITS2 secondary structures. Previous studies have shown that these three species have followed different evolutionary processes compared to other *Avicennia* species [9]. Our results support this theory by confirming their distinct ITS2 structures.

The genus *Avicennia* was separated from Acanthaceae around 41–60 Ma [50]. In this study, it is shown that this genus diverged from the genus *Thunbergia* about 26–36 Ma, during the Oligocene to Eocene period. This is consistent with molecular dating results from related Acanthaceae [55]. Diversification within *Avicennia* began around 26.31 Ma (Oligocene), which corresponds with the global mangrove expansion after Eocene cooling [56].

The divergence of Iranian *A. marina* populations likely occurred during the Pliocene–Pleistocene transition, a period characterized by major fluctuations in sea level [57]. Although the exact timing of this clade’s divergence has not been directly dated, similar patterns observed in other regional mangrove species, such as Rhizophora in the Indo-Pacific [58], suggest that the isolation of the Persian Gulf during Pleistocene glaciations caused genetic differentiation [59]. Thus, Iranian mangroves may represent a peripheral isolated population within the broader *A. marina* complex, which itself began diversifying much earlier, around 13.7 million years ago according to our study.

Biogeographic reconstruction suggests that *Avicennia* originated in Asia and America, with 100% marginal probability support. The ancestral node of *Avicennia* species shows high marginal probability support for an Asian origin, consistent with the inference of primary diversification within this region. The divergence and dispersal patterns observed indicate that *Avicennia* underwent several radiation events, in line with known global climatic and geological shifts that influenced plant distributions [9,60,61].

The Iranian *Avicennia marina* lineage represents a distinct evolutionary trajectory within the species, likely derived from ancestral populations in Asia. This finding is supported by phylogenetic structure and S-DIVA results, which highlight limited subsequent dispersal following the initial colonization of the Middle East. The uniqueness of Iranian populations may reflect geographic isolation and historical climatic fluctuations that promoted local adaptation and divergence. Multiple dispersal events between Asia, the Middle East, and the Americas were inferred (Nodes of 33, 39, 41, 50, and 57), with some nodes suggesting possible long-distance dispersal or stepwise migration. These patterns mirror biogeographic histories seen in other mangrove genera and coastal plant lineages (Duke et al., 1998). While vicariance and limited gene flow may explain regional distinctiveness, the broad distribution of *Avicennia* underscores the genus’s ecological flexibility and dispersal capacity via coastal currents and land connections during periods of lower sea levels.

Biogeographic analysis highlights both the ancient Asian origin of *Avicennia* and the importance of past dispersal and vicariance events in shaping the current distribution of its lineages, especially those found in Iran.

## Conclusion

This study characterized the morphology of *A. marina* in the mangrove forest of Iran. Morphological traits, particularly internode length and pollen characters, support the recognition of a potential new subspecies of *A. marina* in the mangrove forest of Iran. These findings were further moderately supported by the ITS2 secondary structure, we recommend additional studies using alternative DNA barcode markers (e.g., matK, rbcL) or high-resolution genomic approaches (e.g., whole-genome sequencing) to clarify the systematic position of this subspecies in Mangrove forest of Iran.

## Supporting information

S1 Table GenBank accession number species used for the phylogenetic tree drawn. (PDF)
