# Supplementary material for: Morphology and phylogeny of Avicennia marina (Forssk.) Vierh. in Iran
Source: PLoS One. 2026 Jul 7;21(7):e0352461. doi: 10.1371/journal.pone.0352461 (PMC13340853; doi:10.1371/journal.pone.0352461)
Supplement: S1 Table — (PDF) [file pone.0352461.s001.pdf]

Table S1. GenBank accession number species used for the phylogenetic tree drawn

**Species**	**Accession numbers**	**Species**	**Accession numbers**
A. marina	PP237061	*A. officinalis*	MH243944
A. marina	MN883386	*A. integra*	*KX641598*
A. marina	MN883385	*A. marina var. rumphiana*	KX641595
A. marina	MN883384	*A. alba*	MH243936
A. marina	MN883387	*A. officinalis*	MH243946
A. marina	PP087142	*A. officinalis*	MH243947
A. marina	DQ469861	*A. officinalis*	MH243943
A. marina	MH243940	*A. bicolor*	AF365977
A. alba	MH243937	*A. germinans*	AF365979
A. marina subsp. *australasica*	KX641591	*A. schaueriana*	AB861363
A. marina	MH243938	*A. schaueriana*	AB861302
A. marina	KF848262	*A. schaueriana*	AB861240
A. marina	KM652500	*Thunbergia grandiflora*	KX641599
A. officinalis	MH243945		
